# Hybrid resonant cavities: A route towards phase engineered THz metasurfaces

**DOI:** 10.1016/j.isci.2022.104024

**Published:** 2022-03-04

**Authors:** Sukhvinder Kaur, Subhajit Karmakar, Arun Jana, Shreeya Rane, Ravendra Kumar Varshney, Dibakar Roy Chowdhury

**Affiliations:** 1 Department of Physics, Indian Institute of Technology Delhi, Hauz Khas, New Delhi 110016, India; 2 Department of Physics, Ecole Centrale School of Engineering - Mahindra University, Jeedimetla, Hyderabad, Telangana 500043, India

**Keywords:** Photonics, Physics, Radiation physics

## Abstract

Coupled resonant cavities can enable strong photon energy confinement to facilitate the miniaturization of functional photonic devices for applications in designs of sensors, modulators, couplers, waveguides, color filters etc. Typically, the resonances in subwavelength plasmonic cavities rely on the excitation of surface plasmons at specific phase-matching conditions, usually determined by the lattice parameters and constituent material properties. Contrary to this notion, we experimentally demonstrate the control and manipulation of cavity resonances via suitably modifying the split ring resonator geometry in hybrid plasmonic-metasurface (dipole cavity-SRR) configuration without altering the lattice parameters. This results to the excitation of dual resonance peaks. Such dual channel characteristics demonstrate high quality (*Q*) factor, multi-band resonances, not permissible with typical (unhybridized) plasmonic dipole cavities. We envisage such hybrid meta-cavity designs can become important ingredients for futuristic terahertz devices that can hold the key for sixth generation (6G) communications, designer filters, dual channel sensors etc.

## Highlights

• We demonstrate controlling cavity resonances via phase manipulation in THz metasurfaces
• The phase control is achieved through insertion of suitable split ring resonators
• Our demonstrated hybrid cavity structure results in dual channel resonances
• Dual resonances show differential characteristics in terms of intensity & frequency

## Graphical abstract

**Figure:**
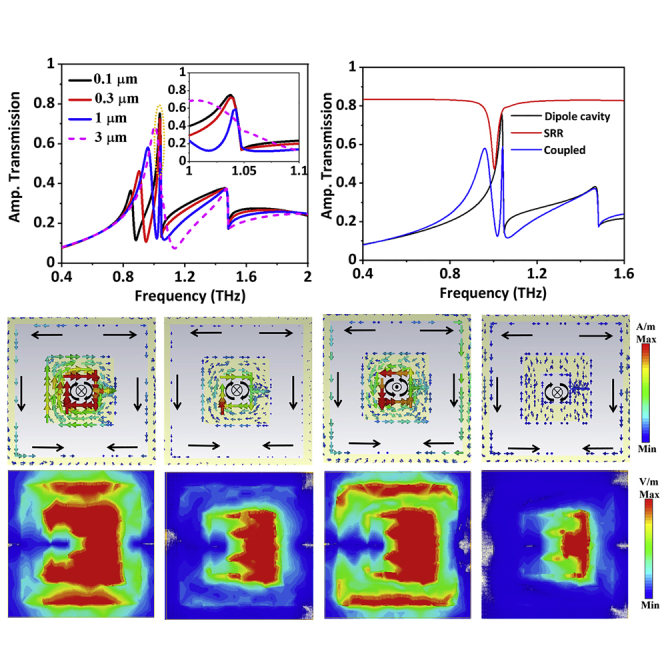

## Introduction

Control over effective electromagnetic responses (viz. dispersion of effective properties of materials; phase, and intensity of electromagnetic radiations) is one of the prerequisites in modern optical/photonic systems across the electromagnetic spectrum, which largely depends on efficient interaction of electromagnetic radiations with the available materials or devices. However, in majority of naturally available materials, there is a persistent lack of several desired properties like strong magnetic response or magnetic coupling at terahertz (THz) or higher frequencies, dispersion of refractive index in spatial domain, phase engineering of transmitted waves, deep-subwavelength high energy confinement with negligible device losses etc (Kadic et al., 2013; Suresh Kumar et al., 2021). To realize such unusual and interesting electromagnetic phenomena, artificially contrived subwavelength metamaterials were proposed, which also have the inherent advantage of its operational ability beyond the diffraction limit. For majority of metasurface devices, fascinating phenomena are brought into the account by engineering the phase of transmitted or reflected waves (be it at its resonance or broadband) (Yu et al., 2011; Mueller et al., 2017; Hung Chu et al., 2016; Sun et al., 2012; Wang et al., 2016; Gholipour et al., 2013). For an instance, split ring resonators (SRRs) were reportedly employed to realize negative permeability and exploiting strong magnetic response in nonmagnetic materials by suitably tuning the phase of the interacting waves (Pendry et al., 1999; Grady et al., 2013). Employing metamaterials/metasurfaces lead to several other fascinating phenomena like negative and anomalous refraction (Smith et al., 2004; Shelby et al., 2001), flat lensing (Pendry, 2000), cloaking (Valentine et al., 2009), subwavelength resolution imaging (Khorasaninejad et al., 2016; Pendry et al., 2012), holography (Larouche et al., 2012; Ni et al., 2013), sensing (Karmakar et al., 2020a; Kaur et al., 2021a) realization of several quantum phenomena (like Fano and toroidal resonance, dark resonating states, electromagnetically induced transparency, bound state in the continuum etc.) in classical domain (Karmakar et al., 2020b, 2021; Kaur et al., 2021b). All these applications are based on engineering the phase of the electromagnetic waves by either abrupt or graded manner in spectral and/or spatial domains. Thus, metasurfaces are one of the viable solutions to tune the phase of transmitted or reflected waves while operating at sub-diffraction regime by modifying its geometry. A contemporary revelation to increase effective transmission efficacy is employing subwavelength periodic holes or cavities in an otherwise opaque metallic sheet. This operates on excitation of surface plasmon resonances at its phase-matching condition and could achieve transmission efficiency larger than unity. Noteworthy, such plasmonic cavities usually violate Bethe’s aperture theory (Bethe, 1944) and increase effective energy confinement in the cavity manifold at its resonances (Ebbesen et al., 1998; Barnes et al., 2004; Martin-Moreno et al., 2001; De Abajo et al., 2005). However, such designs excite purely electric resonances (of dipolar nature) and does not tune magnetic properties and phase (implies dispersion of all the effective parameters of the medium) of the electromagnetic radiation. Moreover, resonance frequencies in such cavity structures cannot be altered by merely varying its geometrical or design parameters (except periodicity and material properties). This is because tuning geometries cannot effectively alter the phase-matching condition to excite surface plasmon resonances at other frequencies. Thus, applicability of such structure is limited by lack of reconfigurable abilities at its resonance frequencies except a few earlier investigations (Gordon et al., 2004; Garcia-Vidal et al., 2005; Jiang et al., 2009; Li et al., 2010). Over the years, despite having numerous intensive investigations, both metamaterials and subwavelength cavities (or holes) were unable to realize its desired potential thoroughly. One suitable solution of this is exploiting the advantages of both the designs, i.e., metamaterials and subwavelength cavities, is to electromagnetically couple them in such a way that at its resonances, the hybrid device can enhance the transmission efficiency (i.e., exhibit properties of subwavelength cavities) and simultaneously tune the properties (dispersion, phase, amplitude etc.) of transmitted electromagnetic radiations (i.e., exhibiting usual metamaterial properties). In recent years, a few demonstrations illustrate combination of metamaterial designs with subwavelength holes. For example, a metamaterial cover without corrugation was proposed to be employed over the subwavelength hole aperture to enhance transmission by minimizing the diffraction losses (Alù et al., 2006). This scheme enhances the transmission through the aperture but the tuning of resonance frequency could not be realized by using the metamaterial. Recently, in another work, a bilayer structure consisting of metallic hole array and complementary metamaterial patch array was also demonstrated to manipulate the transmissions through the subwavelength hole arrays (Banerjee et al., 2020). Although this study could demonstrate significant tuning in resonance intensity, tuning of resonance frequency was bare minimum. Also, excitation of a new transmission peak and its tuning was demonstrated by intruding nanorod(s) in the nanohole array and by varying the length of nanorods operating in the optical regime (Wang et al., 2020a, 2020b). Noteworthy, in all the above demonstrations, metamaterials were employed usually to enhance the transmission efficacy, but the inherent properties of metamaterials (i.e., capability to tune phase and dispersion of the transmitted waves) were not considered, which can possibly significantly modulate the responses of hybrid structures. Thus, a hybrid photonic cavity needs to be explored to exploit additional features in metamaterials' characteristics other than enhanced transmissions through the subwavelength cavities. Therefore, in this work, we have proposed a unique, neoteric hybrid periodic subwavelength device whose unit cell consists of a split ring resonator (SRR) embedded into a subwavelength dipole cavity, thus forming planar cavity metasurfaces. Owing to the capability to engineer phase with metasurfaces, it can be employed very effectively to alter the phase-matching conditions of subwavelength cavities. Although the proposed work shows the passive tuning of resonances, similar tuning can be observed in active fashion through changing the effective capacitance of the split ring resonators by employing light as external stimuli (Roy Chowdhury et al., 2011). Our studied hybrid structure demonstrates two high quality (Q) factor resonance modes with differential frequency and intensity tuning characteristics (implies differential contribution from both the SRR and dipole cavities and SRR-induced phase matching at varied spectral regime). Moreover, strong confinement of magnetic fields at both the resonances (transmission peaks) in these hybrid structures can lead to artificial magnetic features unlike the typical intrinsic subwavelength dipole cavities. The dipole cavities excite purely electric resonances and hence the magnetic characteristics are inconsiderable. However, split ring resonator is well acknowledged for inducing strong magnetic response in nonmagnetic materials by suitably tuning the phase of the interacting waves as demonstrated by Pendry in 1999 (Pendry et al., 1999). A split ring resonator can support the principal eigenmodes with a circular current distribution that gives rise to an induced magnetic moment. Although dipole cavities cannot support artificial magnetism, hybridization with suitably designed SRRs can induce magnetic properties in these typical electric structures, therefore can bring versatility as well as advancement in integrated photonic designs for THz domain.

## Results and discussion

### Proposed structure

**Figure 1:**
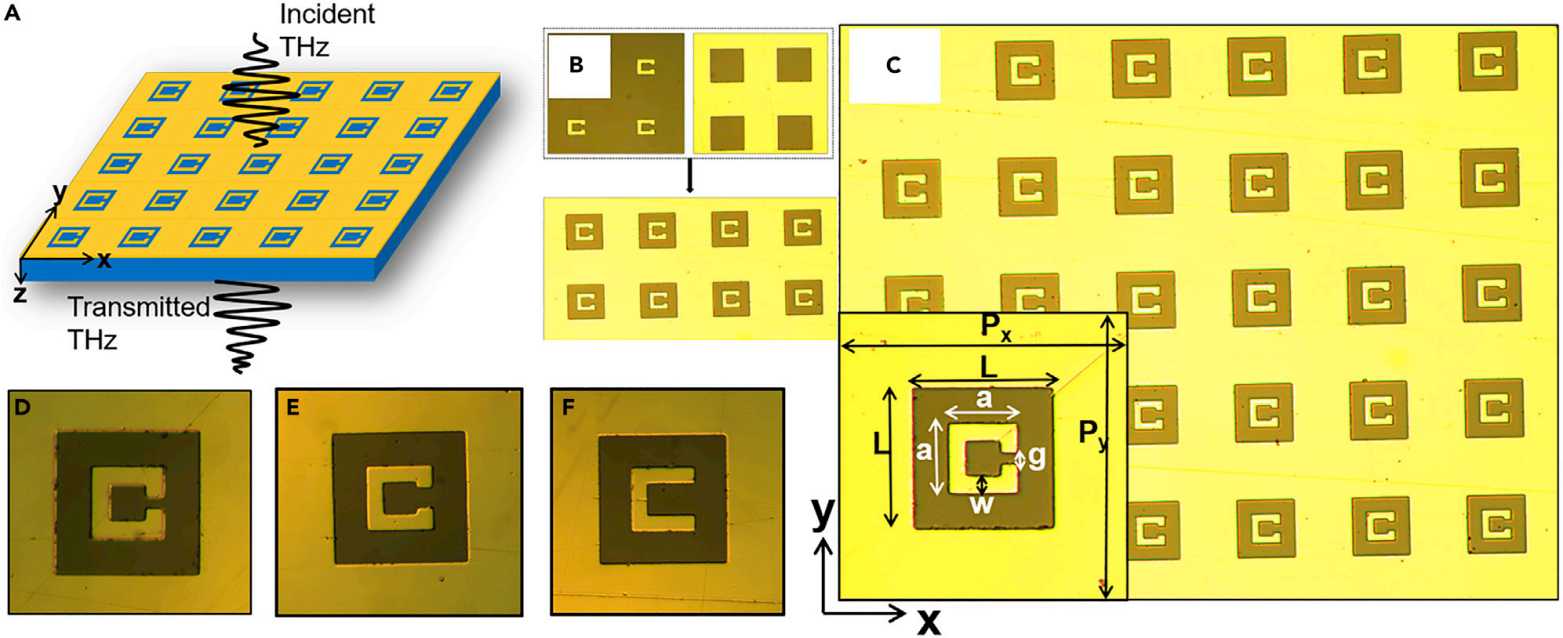
Proposed structure and its optical microscopic images (A) Artistic view of the periodic coupled subwavelength cavity—SRR integrated structure portraying the interaction of incoming THz radiation with the structure. Optical microscopic image. (B) Showing the intrinsic split ring resonator (left), subwavelength cavity (right). (C) Coupled metamaterial array and proposed unit cell (inset) with P_x_ = P_y_ = 83 μm, *L* = 40 μm, *a* = 20 μm, *w* = 5 μm, and varying *g*. (D–F) Unit cell corresponding to different split gap sizes, (D) g = 3 μm, (E) g = 6 μm, (F) g = 10 μm.

The schematic of the proposed structure is shown in Figure 1. An intrinsic silicon wafer (High resistivity of 5,000 Ohm-m) of approximately 530 μm thickness has been used as the substrate. On top of it, a 200 nm thick aluminum (Al) sheet with subwavelength cavities (and each cavity is filled with a single gap split ring resonator with equivalent metal thickness) has been fabricated. Figures 1B–1F show optical microscope images of the uncoupled (intrinsic) as well as coupled samples with different split gaps. The unit cell of the proposed structure is given at the inset of Figure 1C. The periodicity of the proposed structure is considered as 83 μm in both the *x*- and *y*-directions. The square cavity of length (*L*) = 40 μm has been taken into consideration. The SRR dimensions are considered as 20 μm X 20 μm. The split gap (*g*) of the SRR is varied in the present study. Transmission characteristics of the proposed hybrid structure have been investigated both theoretically and experimentally.

### Numerical simulations

The theoretical analysis of the structure has been done by using a finite-difference time-domain (FDTD) solver in commercially available numerical software, CST microwave studio, which solves Maxwell’s equations iteratively with appropriate boundary conditions according to the problem involved. Unit cell boundary conditions are employed to perform the numerical simulations in the transmission mode under normal incidence. Frequency domain solver has been used to numerically simulate the intrinsic/uncoupled as well as coupled hybrid metasurface with varying gap. An adaptive tetrahedral meshing with mesh size λ/4 is employed in all simulations, where λ is the wavelength of the incident radiation. Nearly 30,000 tetrahedron mesh cells have been generated for simulation. The appropriate mesh size is required for fast as well as accurate calculations. The transmission spectra have been recorded for both *x*- and *y*-polarization. Floquet ports are employed for source and detector.

### Sample fabrication and characterization

The fabrications of samples were done by using standard optical photolithography in clean and dry environment on a clean silicon wafer by using an image reversal photoresist. After standard photolithography, metal deposition of 200 nm aluminum was done by direct writing which is followed by lift-off process in the acetone solution resulting in the patterned structures. The samples were characterized by using a photoconductive antenna-based (InGaAs/InP) terahertz time-domain spectroscopy (THz-TDS) (Grischkowsky et al., 1990; Vanderhoef et al., 2014) in a dry environment at room temperature.

### Results

#### For gap (g) > 3 μm

**Figure 2:**
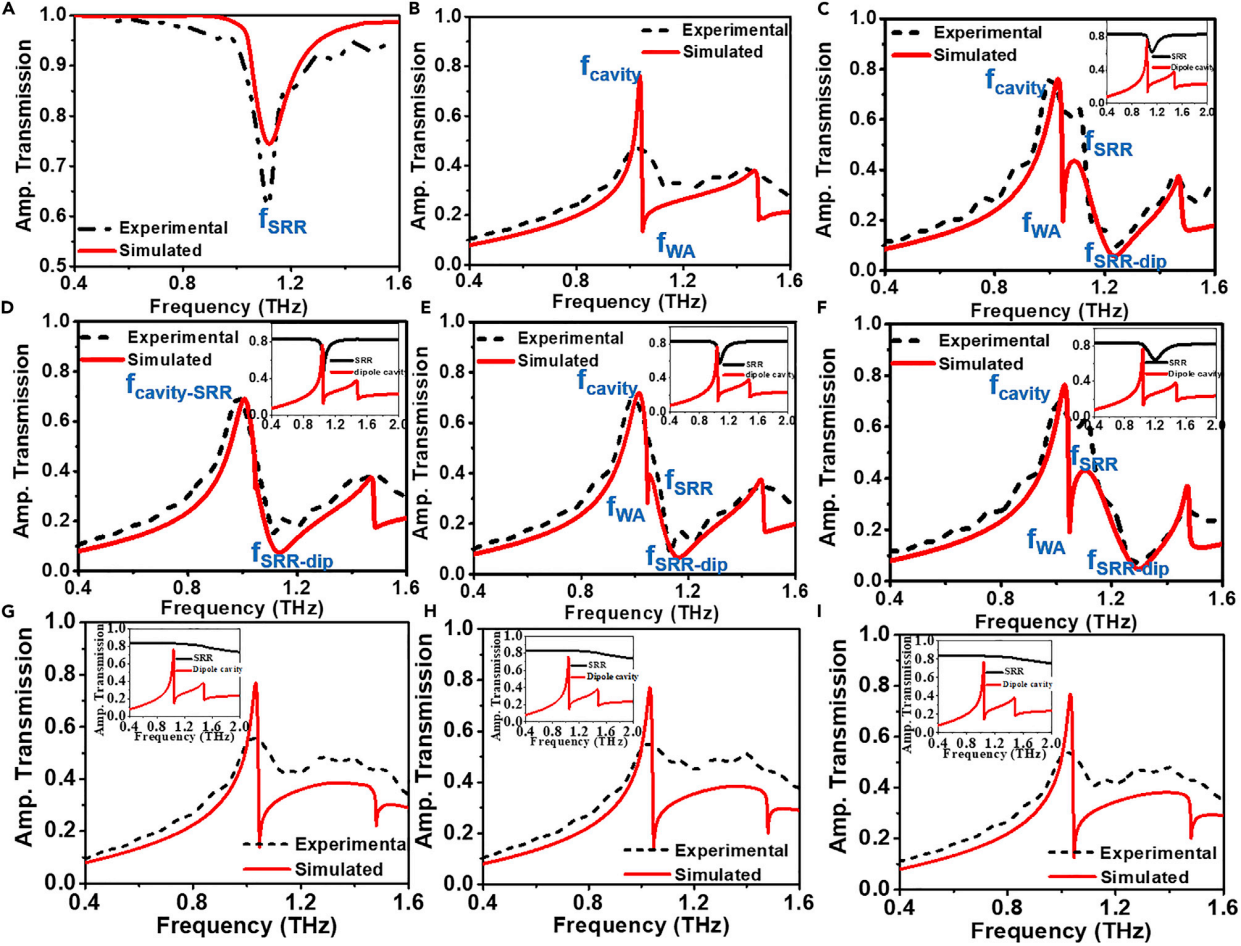
Comparison of experimental (black dashed line) and simulated (red solid line) transmission spectra for different geometries (A) Intrinsic periodic split ring resonators (*g* = 6 μm). (B) Subwavelength cavity. (C–F) Coupled structure corresponding to different gap sizes, viz. (C) *g* = 6 μm, (D) *g* = 3 μm, (E) *g* = 4 μm, (F) *g* = 10 μm with their corresponding intrinsic responses at its insets for *y*-polarization. (G–I) Coupled structure corresponding to different gap sizes, viz. (G) *g* = 3 μm, (H) *g* = 6 μm, (I) *g* = 10 μm for *x*-polarization.

At the beginning, we have investigated the transmission characteristics of the intrinsic (individual) subwavelength cavity and SRR (*g* = 6 μm) independently (in Figures 2A and 2B) and compared the experimental data with the data obtained from the numerical simulations. The intrinsic SRR shows a prominent transmission dip around 1.12 THz (Figure 2A). However, the subwavelength cavity shows the transmission peak at 1.04 THz followed by a transmission dip at 1.05 THz, which can be attributed to Wood’s anomaly (WA) (Jiang et al., 2009; Wood, 1935). The transmission peak at 1.04 THz is due to the excitation of surface plasmon-induced resonance (SPR) (Ebbesen et al., 1998).

To understand the effect of near-field coupling between the SRR and subwavelength cavity, we performed transmission experiments on the coupled hybrid structures with varying gap sizes (*g* = 3, 4, 6, and 10 μm). Figure 2C shows transmission spectra for the coupled SRR (*g* = 6 μm) and subwavelength cavity. The evolution of the $f_{SRR}$ peak at 1.08 THz followed by a transmission dip at 1.22 THz can be comprehended from Figure 2. The formation of the $f_{SRR}$ can be attributed to the integration of split ring resonator in the subwavelength cavity. Generally, the frequency position of the cavity resonance $f_{cavity}$ peak remains fixed if the periodicity and effective parameters (dielectric constant and permeability) of the media remain unaltered (Ebbesen et al., 1998; Jiang et al., 2009). The free-space wavelength of surface plasmons (SPs), *λ*_SP_, in the square lattice is given by (Wood, 1935),

(Equation 1) $$\lambda_{SP}=\;\frac{P}{\sqrt{i^{2}+\;j^{2}}}\;\sqrt{\frac{\varepsilon_{\text{d}}\varepsilon_{\text{m}}\mu_{\text{d}}\mu_{\text{m}}}{\varepsilon_{\text{d}}\mu_{\text{d}}+\;\varepsilon_{\text{m}}\mu_{\text{m}}}}$$

where *P* ($=P_{\text{x}}=P_{\text{y}}$) is the periodicity of the structure, *i* and *j* are integers corresponding to the specific order of the SP and WA mode, $\varepsilon_{\text{d}}$, $\varepsilon_{\text{m}}$, $\mu_{\text{d}}$, and $\mu_{\text{m}}$ are the electric permittivity and magnetic permeability of the substrate and metal, respectively. The resonance takes place at a particular wavelength (Frequency) satisfying the above phase-matching condition and dispersion relation. If the incident wave is propagating along z-direction with k_z_ being its wave vector, then the frequency ω of the oscillations (longitudinal oscillations of surface electromagnetic waves) are connected to its wave vector *k*_x_ by the dispersion relation ω(*k*_x_). Dispersion relation for the interface of metal with dielectric function ($\varepsilon_{1}=\;\varepsilon_{1}^{\prime }+i\;\varepsilon_{1}^{\prime \prime }$) and dielectric with dielectric function ε_2_ is given by (Ghaemi et al., 1998)

(Equation 2) $$k_{x}=\;\frac{\omega }{c}\;{\left(\frac{\varepsilon_{1}\varepsilon_{2}}{\varepsilon_{1}+\varepsilon_{2}}\right)}^{1/2}$$

**Figure 3:**
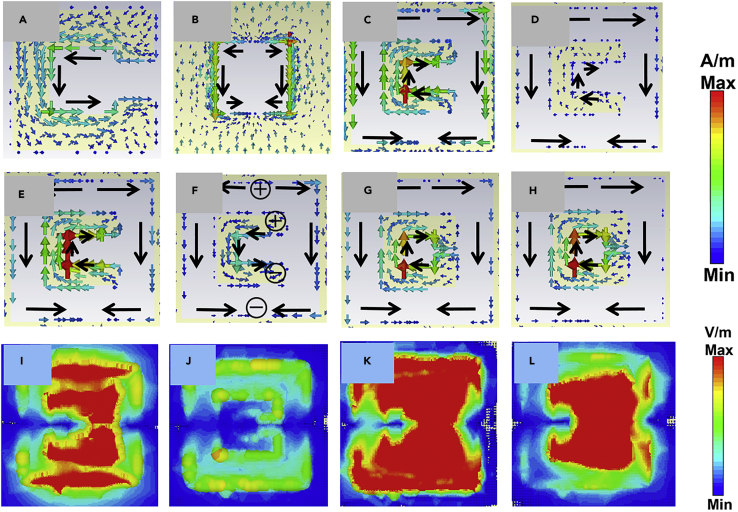
Surface current and magnetic field distribution at various resonance frequencies for various split gaps (in x-y plane) (A and B) Surface current distribution of uncoupled (intrinsic) structures, viz. split ring resonator (g = 6 μm) (A), subwavelength cavity (B). (C–F) Surface current distributions of coupled hybrid structures corresponding to gap size g = 10 μm: (C) $f_{\text{cavity}}$ = 1.03 THz, (D) $f_{\text{SRR}}$ = 1.10 THz, (E) $f_{\text{WA}}$ = 1.05 THz, (F) $f_{\text{SRR}-\text{dip}}$ = 1.3 THz. (G and H) Coupled hybrid structures corresponding to gap size g = 3 μm: $f_{\text{cavity}-\text{SRR}}$ = 1.00 THz (G), $f_{\text{SRR}-\text{dip}}$ = 1.05 THz (H). (I–L) Magnetic field distribution (in x-y) plane: (I) subwavelength cavity; for coupled hybrid structure corresponding to gap size g = 10 μm: (J) $f_{\text{cavity}}$ = 1.03 THz, (K) $f_{\text{SRR}}$ = 1.10 THz; for coupled hybrid structure corresponding to gap size g = 3 μm: $f_{\text{cavity}-\text{SRR}}$ = 1.00 THz (L).

To modify the frequency position of the cavity resonance peak, either the periodicity of the structure or the effective parameters of the medium should be altered. Thus, as seen in Figure 2C, The first (left most) peak ($f_{\text{cavity}}$) at 1.02 THz is excited as the dominant contribution of the subwavelength cavity because this frequency ($f_{\text{cavity}}$) does not significantly overlaps with intrinsic SRR linewidth (1.06–1.21 THz). Thus, frequency position and peak intensity of $f_{\text{cavity}}$ do not alter so much. Noteworthy, outside resonance linewidth, SRR becomes transparent to the incident excitation and does not contribute to the system. Hence, $f_{\text{cavity}}$ shows static characteristics with parametric variations of SRRs (to a certain extent) as shown in Figures 2D–2F). On the other hand, incorporating the SRR, changes the effective parameter (s) (viz. dielectric constant and permeability) of the same media at resonance frequency. The resonance frequency of the split ring resonator is given by the equation (Moghbeli et al., 2018)

(Equation 3) $$f=\;\frac{1}{2\pi \;\sqrt{LC}}=\;\frac{c_{0}}{2\pi }\;\sqrt{\frac{g}{kwa^{2}}}$$

where, a = length of resonator, w = width of SRR, k = dielectric constant of substrate, c_0_ is the speed of light in vacuum and $c_{0}=\;\frac{1}{\sqrt{\varepsilon_{0}\mu_{0}}}$ where, μ_0_ and ε_0_ are the permeability and the permittivity of vacuum and g = split gap. Hence, the resonance frequency varies on varying the split gap size of the resonator. Note that, split gap size affects resonance frequency more dramatically compared to the other geometrical parameters involved in SRR design because of strong confinement of electric energy inside the tiny split gap (Roy Chowdhury et al., 2013). As the resonance frequency varies, it tunes the effective parameter(s) within its resonance linewidth; correspondingly, the phase-matching condition is satisfied at different frequencies other than the intrinsic peak generated due to subwavelength cavities ($f_{\text{cavity}}$) (i.e., surface plasmons generated in subwavelength cavity without having any intrusion in it) for different split gaps, which results in the emergence of a new resonance peak ($f_{\text{SRR}}$) at a different frequency position (implies integrating SRR induces or eases surface plasmon resonance to be excited at different frequencies in subwavelength cavity) other than the intrinsic subwavelength cavity at 1.08 THz in Figure 2C. Within the resonance bandwidth of SRR, the effective area of subwavelength cavities that is exposed to air also decreases, as well as SRR acts as a dissipative media. So, the intensity of the $f_{\text{SRR}}$ tends to be lower than the $f_{\text{cavity}}$. Thus, an entirely different behavioral characteristics is observed at different frequencies (inside and outside SRR linewidth (1.06–1.21 THz)) which successfully justifies the creation of dual resonance peaks: $f_{\text{SRR}}$ and $f_{\text{cavity}}$. The first dip (at 1.05 THz in Figure 2C) corresponds to the Wood’s anomaly ($f_{\text{WA}}$). The corresponding wavelength is given by (Wood, 1935),

(Equation 4) $$\lambda_{WA}=\frac{P}{\sqrt{i^{2}+\;j^{2}}}\sqrt{\varepsilon_{d}}$$

where *P* ($=P_{\text{x}}=P_{\text{y}}$) is the periodicity of the structure, *i* and *j* are integers corresponding to the specific order of the SP and WA mode, and *ε*_d_ is the electric permittivity of the substrate. Its position remains unmodulated or tuned very little as per changes of the effective response of SRR with gap size (only if linewidth overlaps with it (Jiang et al., 2009) and the second dip around 1.22 THz corresponds to response due to SRR resonance $f_{\text{SRR}-\text{dip}}$. The frequency tuning in between coupled and uncoupled resonances is further altered by the near-field coupling (Roy Chowdhury et al., 2015). Figures 2D–2F show the transmission spectra for the split gap (*g*) = 3, 4, and 10 μm, respectively where ($f_{\text{cavity}},\;f_{\text{SRR}},\;f_{\text{WA}},f_{\text{SRR}-\text{dip}})$ appear at 1.03 THz, 1.10 THz, 1.05 THz, and 1.30 THz, respectively for g = 10 μm. Figures 2G–2I show transmission spectra for gap g = 3, 6, and 10 μm with incident polarization along x-direction. It is evident from these plots that addition of subwavelength SRRs does not affect x-polarized light. It can be explained from the inset of Figures 2G–2I. For x-polarization, SRR does not show resonance in frequency range of interest and hence does not affect the effective parameters of the medium resulting in unaffected transmission, whereas the addition of SRR slightly modulate transmission spectra due to increase in metallic area leading toward losses. The surface current distributions corresponding to the intrinsic SRR, and subwavelength cavity are shown in Figures 3A and 3B, respectively. Figures 3C–3F show the surface current distributions for *g* = 10 μm at both the peaks (${\text{f}}_{\text{cavity}},\;f_{\text{SRR}}$): (1.03 THz, 1.10 THz) and dips ($f_{\text{WA}},f_{\text{SRR}-\text{dip}}$): (1.05 THz, 1.30 THz), respectively. For $f_{\text{cavity}}$ (Figure 3C), the contribution of SRR is comparable to the subwavelength cavity contribution but for the second (right) peak ($f_{\text{SRR}}$) (Figure 3D), the contribution of SRR is more in comparison to the subwavelength cavity. The surface current distributions for second dip (Figure 3F) show dominant contribution of SRR. It is clear from the surface current distributions that the SRR actively contributed in the hybridization of the resonances. Hence, the SRR affects the surroundings (phase-matching condition) of the subwavelength cavity and responsible for the creation/manipulation of the second resonance peak. Figure 3I shows magnetic field distribution for subwavelength cavity and Figures 3J and 3K show magnetic field distribution for *g* = 10 μm at both the peaks ($f_{\text{cavity}},\;f_{\text{SRR}}$): (1.03 THz, 1.10 THz). For subwavelength cavity, the magnetic field is confined only at the periphery of the cavity and there is no magnetic field inside the cavity. On integrating the SRR inside the cavity induces magnetic fields inside the cavity also. For $f_{\text{cavity}}$ = 1.03 THz, the magnetic field is induced due to combination of both the cavity and SRR while for $f_{\text{SRR}}$ = 1.10 THz, the magnetic field is concentrated at SRR only which again confirms the dominant contribution of SRR in generating second peak.

#### For 2.3μm ≤ g ≤ 3 μm

**Figure 4:**
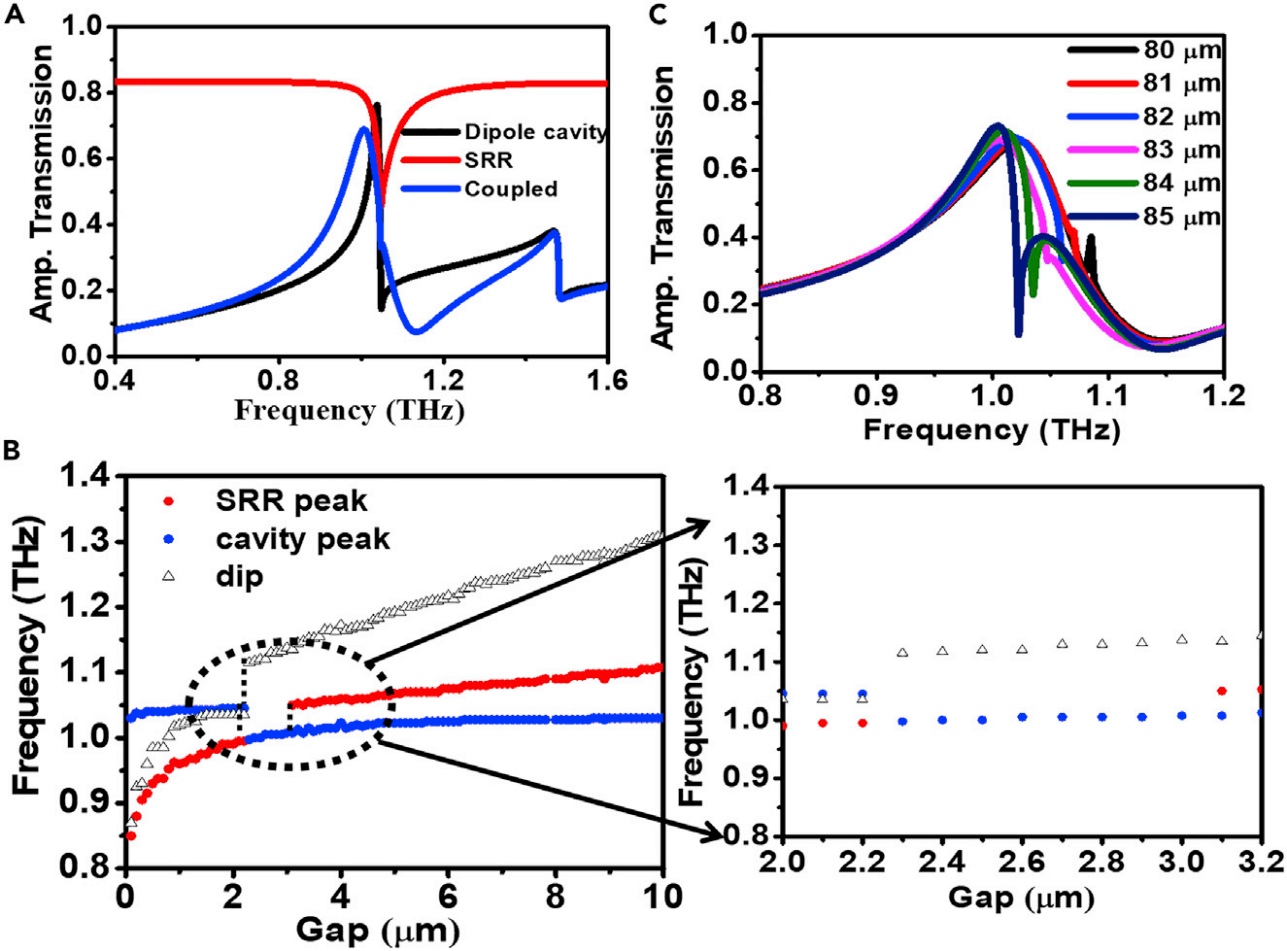
Transmission characteristics of the structure at crossing point and graphical visualization of crossing point (A) Transmission spectra of coupled and uncoupled structure for gap size g = 3 μm. (B) Variation in frequency position of both the peaks and dip with gap size. (C) Transmission spectra corresponding to different periodicity for g = 3 μm.

Figure 2D shows the comparison of experimental and simulated transmission spectra for the coupled structure with *g* = 3 μm and Figures 3G and 3H show the surface current distribution corresponding to the resonance peak ($f_{\text{cavity}-\text{SRR}}$) at 1.00 THz and dip $f_{\text{SRR}-\text{dip}}$ at 1.05 THz and Figure 3L shows the magnetic field distribution corresponding the resonance peak. It is evident from the Figure 2D that there is single resonance peak ($f_{\text{cavity}-\text{SRR}}$) for *g* = 3 μm despite the presence of SRRs. Here, the overall tuning of frequency position, intensity, and linewidth of resonance peak (compared to the intrinsic case as shown in Figure 4A) is due to the overlapping of the response of the SRR to the subwavelength cavity. Here, the linewidth of SRR overlaps with the linewidth of resonance peak of intrinsic subwavelength cavity. Thus, unlike the above cases, the effective phase-matching condition (Equation 1) in dispersion relation for surface plasmons satisfies at a single frequency rather than two different frequencies, which results in a single transmission peak at *g* = 3 μm. Figure 4B shows the frequency shift of both the peaks ($f_{\text{cavity}}$, $f_{\text{SRR}}$) and dip ($f_{\text{SRR}-\text{dip}}$) with the split gap of SRR. Characteristic frequency crossing is observed clearly from Figure 4B. It is clear from Figure 4B that for 2.3μm ≤ g ≤ 3 μm window, we have only peak in transmission spectra. At g = 2.3 μm, the resonance dip of SRR coincides with the Wood’s anomaly dip and hence changes the effective parameters of the structure at that particular frequency. It tunes the effective parameters at that frequency in such a way that the wavelength satisfying the phase-matching condition for localized surface plasmons for SRR coincides with the phase-matching condition for the dipole cavity and hence results in a single peak at that frequency. Owing to the dissipative behavior of the split ring resonator, the intensity of the peak changes whereas the shift in dipole cavity resonance frequency is due to the reason that the SRR changes the effective parameters of the structure within its resonance linewidth. The resonance linewidth of the SRR coincides with the linewidth of the dipole resonance and hence, the effective parameter in that linewidth changes which modifies the wavelength satisfying the phase-matching condition in Equation 1. Hence, the resonance frequency of dipole cavity changes. After crossing g = 2.3 μm, the SRR dip crosses the Wood’s anomaly dip and again results in a new peak, but this peak is relatively much smaller and due to losses in the metal this peak cannot be detected. As the gap increases, the intensity of the peak increases and after crossing g = 3 μm, the peak can be seen in the transmission spectra. The newly generated peak shows the dominating contribution of SRR. Therefore, for gap sizes other than 2.3μm ≤ g ≤ 3 μm, the phase-matching condition satisfies at two different frequencies for subwavelength cavity and SRR and results in generation of another resonance peak. With tuning the split gap of SRR, line shape of resonance peaks changes. At g = 2.3 μm, the resonance peak transfers from diffraction regime to sub-diffraction regime. The dependency of the crossing point on the lattice mode has been investigated by varying the periodicity of the structure at g = 3 μm. Figure 4C shows the transmission spectra for different periodicities. On varying the periodicity of the structure, λ_SP_ changes and the phase-matching conditions are satisfied at different frequencies. In this case, the phase relation for surface plasmons satisfied at two different frequencies: one due to subwavelength cavity and the other due to SRR again, hence resulting in two resonance peaks. Therefore, the split gap corresponding to the frequency crossing point depends upon the periodicity of the complex hybrid structure.

#### For gap (g) < 2.3 μm

**Figure 5:**
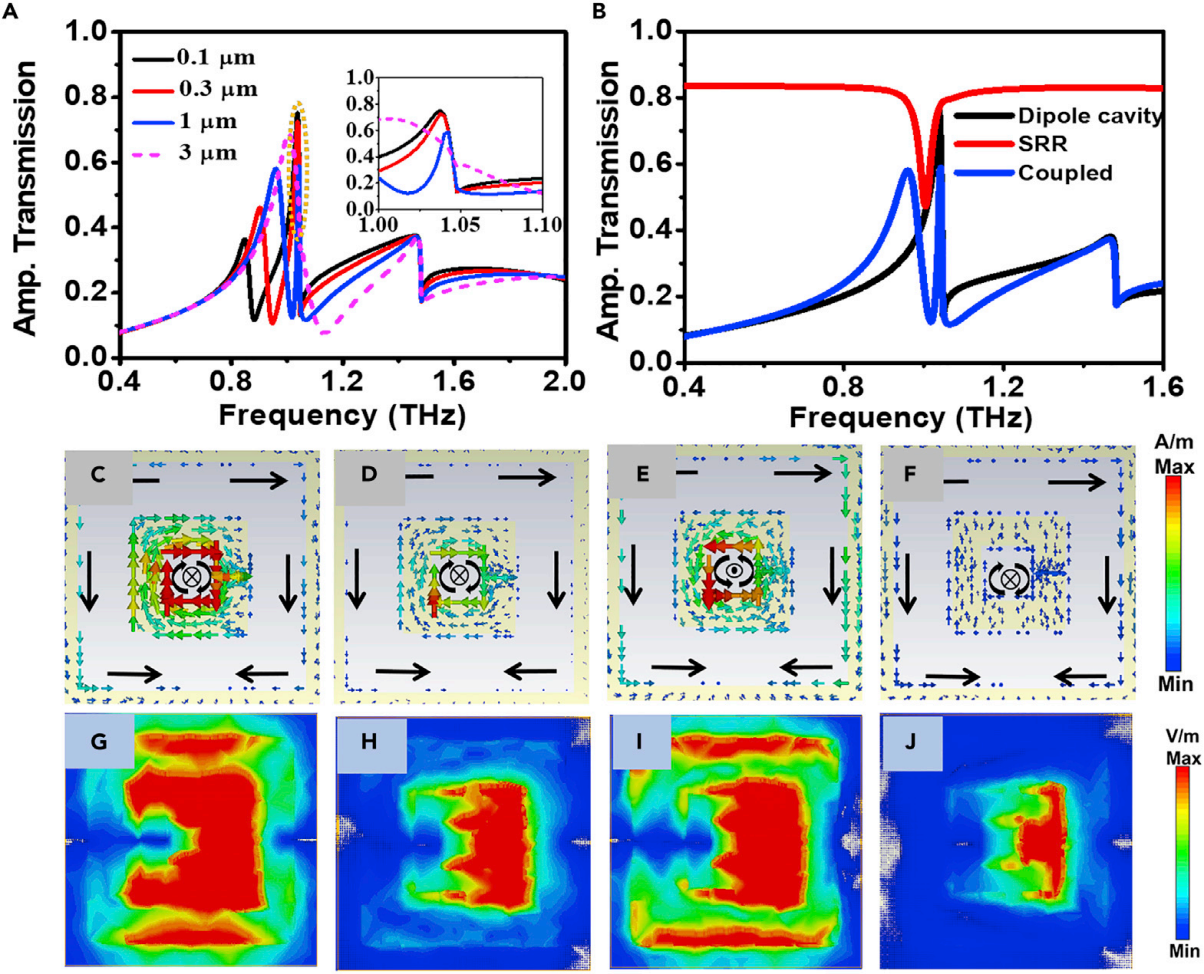
Transmission characteristic, surface current, and electric field distribution for split gap sizes g ≤ 3 μm (A) Transmission spectra for coupled hybrid structure corresponding to different gap sizes for g ≤ 3 μm. (B) Transmission spectra of coupled and uncoupled structure for gap size g = 1 μm. (C–F) Surface current distribution for gap g = 1 μm: (C) $f_{\text{SRR}}$ = 0.96 THz, (D) $f_{\text{SRR}-\text{dip}}$ = 1.02 THz, (E) $f_{\text{cavity}}$ = 1.04 THz, (F) $f_{\text{WA}}$ = 1.07 THz. (G–J) E-field distribution for g = 1 μm: (G) $f_{\text{SRR}}$ = 0.96 THz, (H) $f_{\text{SRR}-\text{dip}}$ = 1.02 THz, (I) $f_{\text{cavity}}$ = 1.04 THz, (J) $f_{\text{WA}}$ = 1.07 THz.

On further decreasing the gap, the broadening of the resonance peak reduces due to its transition into sub-diffraction regime and we obtain a narrow transmission peak. It is evident from Figure 4B that for lower values of the gap, the frequency position of the $f_{SRR}$ peak changes significantly in comparison to the $f_{\text{cavity}}$, which has dominant contribution from subwavelength cavities. Hence, tuning of geometric parameters of SRRs allowed the modulation of frequency position ($f_{\text{SRR}}$) at large spectral range (up to 0.26 THz with variation of split gap from 200 nm to 10 μm). Figure 5A shows the transmission spectra for the varying split gap and Figure 5B illustrates the comparison of transmission spectra for intrinsic SRR and subwavelength cavity with the coupled structure for g = 1 μm. Figures 5C–5F show the surface current distribution for g = 1 μm at both the peaks and dips ($f_{\text{SRR}},f_{\text{SRR}-\text{dip}},\;f_{\text{cavity}},\;f_{\text{WA}}$). Figures 5G–5J represent the corresponding electric field distributions. Careful observation of surface currents and electric field distributions reveals the nature of coupling in between the SRR and the subwavelength cavity. It is evident from the surface current distribution that the phase of the new resonance peak changes as it enters the sub-diffraction regime after the crossing point. It is clear that the contribution of SRR is larger compared to subwavelength cavity for the $f_{\text{SRR}}$ whereas for $f_{\text{cavity}}$, both the subwavelength cavity and SRR has approximately equal contribution. The presence of SRR does not alter significantly the position of the $f_{\text{cavity}}$ because SRR is transparent to incident radiation for frequencies other than the resonance frequency of SRR. Therefore, it does not affect the effective parameter(s) and $f_{\text{cavity}}$ remains unaltered. However, before (after) crossing window (i.e., at 2.3μm ≤ g ≤ 3 μm), $f_{\text{SRR}}$ appears at lower (higher) frequencies than $f_{\text{cavity}}$ signifying that we can effectively achieve resonance peaks with differential characteristics (dominant subwavelength cavity (electric) and SRR (magnetic) behavior) at hybridized modes with different photon energies (implies at different frequencies). Furthermore, it is evident from Figures 5D and 5H that the dip ($f_{\text{SRR}-\text{dip}}$) at 1.02 THz is solely due to resonance of SRR as there is no contribution of subwavelength cavities.

The blue shifting of the coupled resonance dip in all three cases with respect to their intrinsic resonance dip can be attributed to the near-field coulomb interaction effect (Roy Chowdhury et al., 2015). Moreover, the blue shifting of resonance dips ($f_{\text{SRR}-\text{dip}}$) for coupled cases with respect to uncoupled/intrinsic cases increases with the increase in split gap size. Figure 3F shows the surface current polarity for both the SRR and subwavelength cavity. As we increase the split gap of SRR, the effective distance between the charges accumulated at the split gap and subwavelength cavity decreases. Both SRR and subwavelength cavity have same polarity charges on same side and the decrement in the effective distance leads toward the increase in coulomb repulsion between SRR and subwavelength cavity resulting in the blue shift of resonance dip. Additionally, it is also observed that the new transmission peak ($f_{\text{SRR}}$) gets blue-shifted on increasing the split gap of the SRR due to the decrement of effective capacitance of the system. This blue shifting can be attributed to the decrement of the effective capacitance on increasing the split gap of the SRR (Aydin et al., 2005).

### Conclusion

In summary, we have devised a hybrid design where suitable metasurface (viz. SRR) is introduced into periodically arranged subwavelength cavities. By satisfying phase-matching conditions at different frequencies with variation of split gaps in SRRs, our demonstrated cavity structure can elucidate simultaneously metamaterial properties (strong magnetic coupling) and enhanced transmissions at the newly generated resonances (i.e., transmission peaks generated due to coupling of metasurfaces with subwavelength cavities). The observed dual resonances (transmission peaks) exhibit differential properties with respect to each other in terms of intensity and frequency tunability. Moreover, our demonstrated hybrid metasurface paves the way to satisfy phase-matching conditions at different frequencies without changing the constituting material properties or lattice constant. Thus, our scheme can be a cornerstone to remove long-standing limitations like tuning surface plasmon-mediated transmission positions (resonance frequencies) by simply changing geometries (either active or passive manner) of the device, and introducing magnetic properties in subwavelength cavities which can be highly promising in several futuristic applications in spectral enhancement, frequency filtering, efficient phase engineering, sensing, communications, detectors, filters etc. in the THz regime.

### Limitations of study

The current study is based on passive mode of operation, this means it does not involve the active tuning of resonance response. Passive metasurfaces imply that the responses of the fabricated metasurfaces cannot be altered by external means. However, in future, this idea can be extended to include active tuning employing external stimulus (ex. Voltage, light, temperature etc) in order to enhance suitability in practical applications.

## STAR★Methods

### Key resources table

**Table:** 

REAGENT or RESOURCE	SOURCE	IDENTIFIER
**Other**		

AZ5214E	Merck Performance Materials GmbH	CAS# 91-04-3
AZ726MIF	Merck Performance Materials GmbH	CAS# 75-59-2
Aluminum	Testbourne Ltd	CAS# 7429-90-5
Silicon wafer	University wafer	CAS# 7440-21-3
Acetone	Merck Performance Materials GmbH	CAS# 67-64-1
Isopropanol	Merck Performance Materials GmbH	CAS# 67-63-0

**Software and algorithms**		

Origin 8.5	Origin lab	https://www.originlab.com/
CST Microwave studio	3ds simulia	https://www.3ds.com

### Resource availability

#### Lead contact

Further information and requests for resources should be directed to and will be fulfilled by the lead contact, Professor Dibakar Roy Chowdhury (dibakar.roychowdhury@mahindrauniversity.edu.in)

#### Material availability

This study did not generate new materials.

#### Data and code availability

• Data: The data that support the findings of this study are available from the authors on reasonable request. See author contributions for specific datasets.
• Code: This paper does not report code.
• For any additional questions or information please contact the lead contact.

### Method details

#### Experimental procedures

##### Device fabrication

The samples were fabricated on intrinsic silicon substrate by using optical photolithography in clean and dry environment by an image reversal photoresist AZ5214E for high resolution images. The photoresist was pre-baked at 110°C for one minute and then after cooling it down, the second photoresist bake (Post exposure bake) was done at 110°C for two minutes. AZ726MIF developer was used for development time of 30 seconds. After the standard photolithography, metal deposition of 200 nm aluminium was done by direct writing using Heidelberg tool followed by lift off process in the acetone solution resulting in the patterned structures (Figure 1). Various sets of the proposed hybrid structures were fabricated. To understand the intrinsic response of subwavelength cavity and SRR, the intrinsic (uncoupled or individual) subwavelength cavity and SRR were fabricated on an intrinsic silicon wafer. Additionally, we fabricated four coupled (complex hybrid metasurface) samples with different split gap sizes.

##### Device characterization

The samples were characterized by using a photoconductive antenna based (InGaAs/InP) Terahertz time domain spectroscopy (THz-TDS) in a dry environment at room temperature. A femtosecond laser (1560 nm, 100 MHz, ∼60 fs) has been used to generate terahertz pulses with a spectral resolution of around 67 GHz using the Teraflash THz source (Toptica Photonics). The laser pulse splits into two parts, one part traveling towards the terahertz emitter and the other part towards the detector. The detector and emitter contain a 25 μm dipole antenna and a 100 μm strip-line antenna, respectively, both consisting of InGaAs/InP photoconductive switches. The terahertz pulses reach the receiver after transmitting through the samples. These transmitted THz pulses were detected in the time domain using the pump-probe principle. A blank piece of identical silicon substrate was used as a known reference material and transmitted THz pulses through the blank substrate were used as reference signals. The frequency-domain spectrum is then extracted through fast Fourier transformation (FFT) of the measured time-domain transmitted terahertz pulse. To obtain the amplitude transmission of a sample, the spectrum transmitted through the sample were normalized with the spectrum transmitted through the reference in order to capture the responses.

### Quantification and statistical analysis

This study does not include statistical analysis or quantification.
